# Assessing the Performance of Clinical Natural Language Processing Systems: Development of an Evaluation Methodology

**DOI:** 10.2196/20492

**Published:** 2021-07-23

**Authors:** Lea Canales, Sebastian Menke, Stephanie Marchesseau, Ariel D’Agostino, Carlos del Rio-Bermudez, Miren Taberna, Jorge Tello

**Affiliations:** 1 Department of Software and Computing System University of Alicante Alicante Spain; 2 MedSavana SL Madrid Spain

**Keywords:** natural language processing, clinical natural language processing, electronic health records, gold standard, reference standard, sample size

## Abstract

**Background:**

Clinical natural language processing (cNLP) systems are of crucial importance due to their increasing capability in extracting clinically important information from free text contained in electronic health records (EHRs). The conversion of a nonstructured representation of a patient’s clinical history into a structured format enables medical doctors to generate clinical knowledge at a level that was not possible before. Finally, the interpretation of the insights gained provided by cNLP systems has a great potential in driving decisions about clinical practice. However, carrying out robust evaluations of those cNLP systems is a complex task that is hindered by a lack of standard guidance on how to systematically approach them.

**Objective:**

Our objective was to offer natural language processing (NLP) experts a methodology for the evaluation of cNLP systems to assist them in carrying out this task. By following the proposed phases, the robustness and representativeness of the performance metrics of their own cNLP systems can be assured.

**Methods:**

The proposed evaluation methodology comprised five phases: (1) the definition of the target population, (2) the statistical document collection, (3) the design of the annotation guidelines and annotation project, (4) the external annotations, and (5) the cNLP system performance evaluation. We presented the application of all phases to evaluate the performance of a cNLP system called “EHRead Technology” (developed by Savana, an international medical company), applied in a study on patients with asthma. As part of the evaluation methodology, we introduced the Sample Size Calculator for Evaluations (SLiCE), a software tool that calculates the number of documents needed to achieve a statistically useful and resourceful gold standard.

**Results:**

The application of the proposed evaluation methodology on a real use-case study of patients with asthma revealed the benefit of the different phases for cNLP system evaluations. By using SLiCE to adjust the number of documents needed, a meaningful and resourceful gold standard was created. In the presented use-case, using as little as 519 EHRs, it was possible to evaluate the performance of the cNLP system and obtain performance metrics for the primary variable within the expected CIs.

**Conclusions:**

We showed that our evaluation methodology can offer guidance to NLP experts on how to approach the evaluation of their cNLP systems. By following the five phases, NLP experts can assure the robustness of their evaluation and avoid unnecessary investment of human and financial resources. Besides the theoretical guidance, we offer SLiCE as an easy-to-use, open-source Python library.

## Introduction

Over the last decades, health care institutions have increasingly abandoned clinical records in paper form and have started to store patients’ longitudinal medical information in electronic health records (EHRs). EHRs are widely available and capture large amounts of valuable clinical information from medical backgrounds, examinations, laboratory testing, procedures, and prescriptions [1]. While some clinical data are codified in the structured fields of EHRs, the great majority of relevant clinical information appears embedded within the unstructured narrative free-text [2]. In this free-text section, physicians write down their routine evaluation of the patient and thereby offer a window into real-world clinical practices [3,4].

The resulting exponential growth of digitized data on real-world clinical practice has given rise to specialized research fields such as clinical natural language processing (cNLP) [5,6], which aims at exploring the clinically relevant information contained in EHRs [7-9]. The importance and complexity of improving cNLP systems has given rise to a strong engagement among researchers in developing methods capable of doing so [10-16]. This resulted in improved cNLP systems that have dramatically changed the scale at which information contained in the free-text portion of EHRs can be utilized [17-20] and has provided valuable insights into clinical populations [21-27], epidemiology trends [28-30], patient management [31], pharmacovigilance [32], and optimization of hospital resources [33].

However, there is a lack of guidance on how to evaluate those cNLP systems [34]. Although some ground-breaking work was done by Biber [35] and Paroubek et al [36], who analyzed the representativeness in linguistic corpora and the quantity and quality of annotations needed to establish a representative gold standard, hardly any proposal exists for an end-to-end evaluation methodology of cNLP systems. Criteria for the evaluation of cNLP systems were provided by Friedman and Hripcsak [37] and, 10 years later, Velupillai et al [38]. Those are actionable suggestions to improve the quality of cNLP system evaluations. Based on their judgment, the provision of details about the number of domain experts who participated in the creation of the reference standard, mentions of the sample size, defining the objective of the study, and the presentation of performance measure CIs were deemed relevant aspects that provide robustness to cNLP evaluations [35-37]. Such criteria are key to advancement in cNLP [37] because of the direct and existential impact these systems have on understanding patients and diseases [39].

A crucial point for the evaluation of a cNLP system is the availability of benchmark data sets in a specific language based on real EHRs. Although many corpora for the medical domain are available in English, they are scarce or nonexistent for other languages. As a consequence, many benchmarks have been designed a priori for clinical publications and are not real EHRs [40]. The downside of this practice is that some important values present in real EHRs are not contained in artificial EHRs. For example, the validation of artificial data sets may not include variables or concepts of the pathology of interest or research objective. Furthermore, real-world data sets entail misspellings, acronyms, and other particularities of the free-text narratives of patients’ EHRs, which can be taken into account in the validation process, thereby providing a much more accurate and generalizable evaluation of the cNLP system [41]. Obviously, the use of actual EHRs obliges researchers to implement the necessary steps and tools to guarantee the confidentiality and security of the data, in compliance with hospital ethics committees, national and international regulations, and pharmaceutical industry policies.

Here, we propose a language-independent evaluation methodology that can help researchers to overcome some of the mentioned obstacles when evaluating their cNLP system. Our objective is to provide a state-of-the-art methodology for the evaluation of cNLP systems, thereby guiding researchers in the field of natural language processing (NLP) in this complex process to ensure the robustness and representativeness of the system’s performance metrics. The proposed evaluation methodology is the result of our experiences developing cNLP evaluations in real use-cases dealing with heterogeneous EHRs focusing on a wide range of pathologies from one or several hospitals in different countries.

## Methods

**Figure 1:**
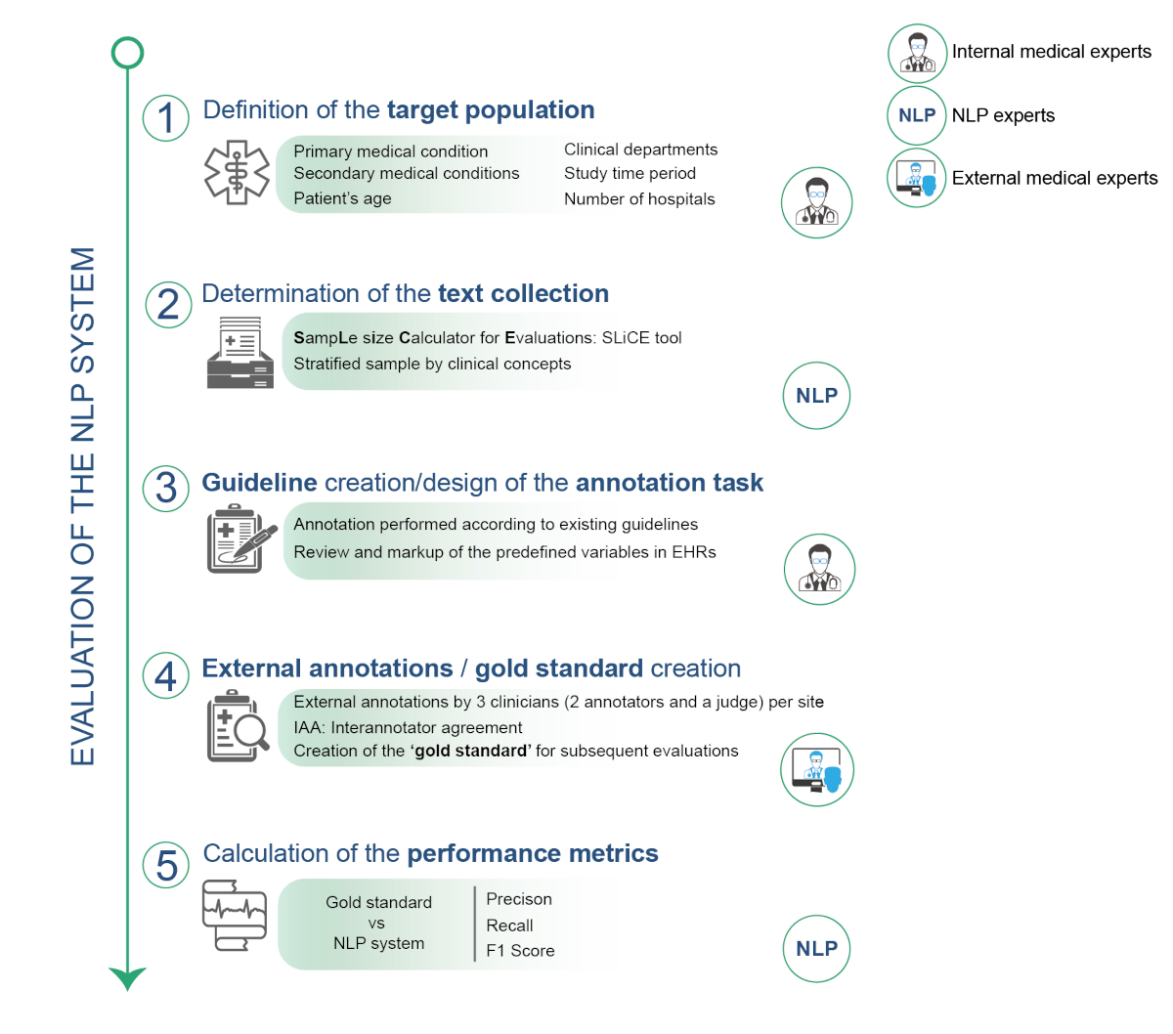
The proposed evaluation methodology consists of five phases that guarantee the evaluation of a clinical natural language processing system against a gold standard providing unbiased performance metrics. NLP: natural language processing, EHR: electronic health record.

Our evaluation methodology is a set of methods and principles used to perform a cNLP system evaluation, which extends from the establishment of the reference standard to the measurement and presentation of the evaluation metrics. It consists of five phases : (1) definition of the target population, (2) statistical document collection, (3) design of the annotation guidelines and annotation project, (4) external annotations and gold standard creation, and (5) cNLP system performance evaluation (Figure 1).

In the following paragraphs, we present the five phases of the proposed evaluation methodology in the context of cNLP systems. However, this approach is not limited to cNLP systems and the phases can be adapted to perform equally useful evaluations of nonclinical NLP systems.

### Phase 1: Definition of the Target Population

The target population is defined by sets of nonlinguistic and linguistic characteristics. Nonlinguistic characteristics of the target population are, for example, the type of hospitals that participate in the evaluation, as this defines the clinical departments commonly in charge of those patients, or factors such as patient age (eg, patients under 18 years of age for a pediatric disease) or gender (eg, men for studying prostate cancer). Linguistic characteristics on the other hand are related to the actual written content in an EHR such as mentions of the primary and secondary medical conditions being evaluated. It is highly recommended to consider secondary medical conditions since they help to determine the criteria of sampling. A list of questions related to the nonlinguistic and linguistic characteristics, which needs to be answered by the responsible medical experts, helps to identify the scope of the cNLP system evaluation, the requisites for sampling, and the sample size:

- Patient age: is the patient’s age relevant in the studied pathology?
- Hospitals: which hospitals will participate in this evaluation?
- Clinical departments: are there any clinical departments related to the disease that are relevant for this evaluation?
- Time: is there a period of time in which the evaluation should be carried out? (study period)
- Primary medical condition (primary variable): which disease or primary medical condition will be evaluated?
- Secondary medical conditions (secondary variables): which other medical conditions or medical evaluations (eg, symptoms, signs, treatments, or tests) will be considered?

### Phase 2: Statistical Document Collection Using the Sample Size Calculator for Evaluations

Determining the amount of data needed to capture enough linguistics to be statistically robust as well as selecting the sample to produce consistent performance measures, has been an open question in NLP research for more than a decade [35-37]. In our evaluation methodology, a linguistic event refers to a particular clinical concept mentioned in EHRs such as a disease, a symptom, or a sign. Thus, the aim of phase 2 is to build a corpus which represents the characteristics of the population as closely as possible by combining an in-house software tool called Sample Size Calculator for Evaluations (SLiCE) and stratified sampling.

#### SLiCE

SLiCE is a publicly available software [42] developed by Savana, an international medical company, that enables users to estimate the minimum sample size required to obtain robust metrics of reading performance, whereby robustness is determined by predefining the CI and level. The method was designed using the standard metrics commonly applied in NLP system evaluations: precision (P), recall (R), and F1-score [43]. The input parameters of SLiCE are (1) the desired confidence level (1-alpha), (2) the CI width, (3) expected values of precision and recall, (4) the frequency of the linguistic event to evaluate, and (5) whether this frequency has been calculated “internally” or “externally.” The output of SLiCE contains the sample size as well as the number of positive and negative samples required to ensure the CI for the linguistic events evaluated. The final number of documents to manually annotate is to be shared equally among the participating hospitals in case of a multisite evaluation.

The fundamentals of SLiCE are based on the sample size determination method [44] for proportions [45] and the expected occurrence rate (prevalence) of a linguistic event in the total population. The method consists of fixing a confidence level and a CI to calculate the sample size required to achieve the desired CI. In our proposal, the Clopper-Pearson approach is employed for CI calculation [45] since it is a common method for calculating binomial CI. Under the Clopper-Pearson approach, the lower and upper confidence limits are determined by:

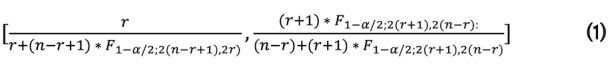



where *n* is the number of trials (sample size), *F* is the F-Snedecor distribution, *r* is the number of successes, and α is the significance level (eg, 5%).

The proposed method is applicable when the objective is to assess a linguistic event or a set of linguistic events. Consequently, the definition of the target population is key to applying SLiCE since the calculation of the prevalence of the event in the target population is a requirement.

**Figure 2:**
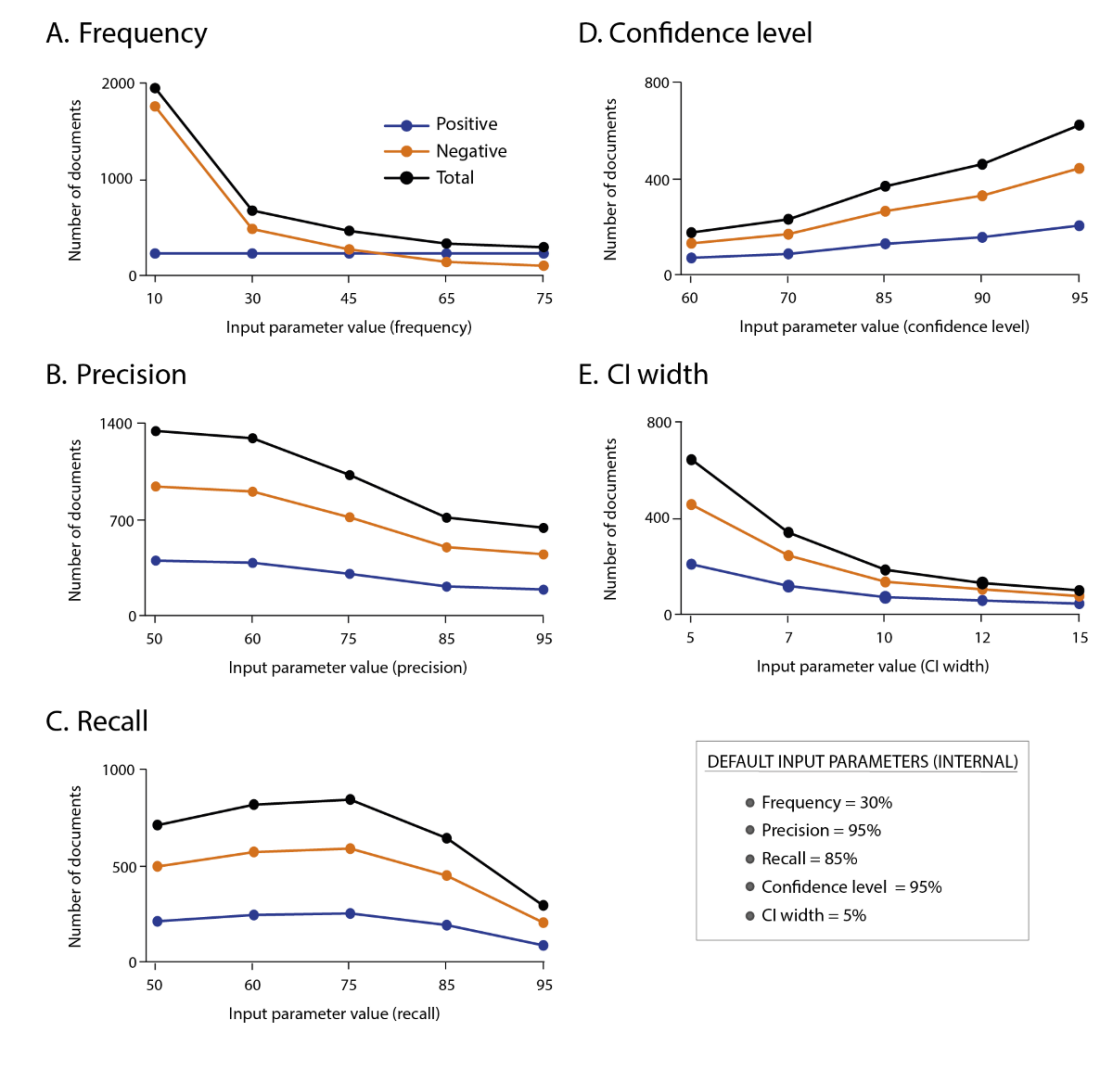
Analysis of SLiCE (Sample Size Calculator for Evaluations) outputs according to changes in input parameters and their impact on the number of documents to be selected for the gold standard.

The expected values of precision and recall represent values that are considered achievable by the system. Care should be taken not to overestimate the performance of the system by introducing values higher than 90% when the actual performance is below. This would result in a very small sample size and, consequently, final metrics that are not very robust. If our system achieves values in the evaluation that are far from the expected ones, the probability of complying with the CI is low. Therefore, we recommend applying realistic values of P and R (around 80%) to ensure the robustness of the final metrics. The impact of the frequency of a main variable is the most influential input as more negative examples are needed in case of low frequency to guarantee a representative sample. To achieve a more robust cNLP system evaluation, more documents would need to be annotated. On the contrary, if high recall and precision are expected, the total number of documents to verify this expectation is lower than when low recall and precision are expected (Figure 2).

SLiCE has two additional options which are “internal” and “external.” When “internal” is selected (default), the occurrence rate of the main variable needs to be calculated. To achieve that, the prevalence of this linguistic event can be calculated using the data provided in each hospital. Thus, for each hospital participating in a study, the frequency using the following formula can be calculated:

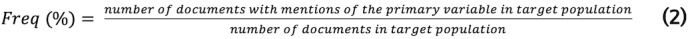



In the case of developing the evaluation for several hospitals, the final frequency is the average of the occurrence rate of the main variable in each hospital. Poor prevalence variables might require a very large number of documents to be annotated, which is not feasible in practice. However, the prevalence could be measured not from an entire database, but from a subset of relevant EHRs (eg, only a specific department).

When a set of linguistic events (eg, clinical concepts) is evaluated, SLiCE needs to be applied to each clinical concept that defines the target population to ensure the expected CI for all the clinical events. However, this may not always be possible due to time and budget restrictions associated with an evaluation. For this reason, our methodology proposes to apply SLiCE at least for the primary variable defined in the target population. Consequently, for the secondary variables, the sample size does not need to be calculated because they depend on the sample size calculated for the primary variable.

It is important to note that the parameters of the calculator should be decided by the medical experts together with NLP experts in charge of the study based on their expectations regarding the performance of the system. A detailed explanation about the SLiCE algorithm can be found in Multimedia Appendix 1, and a guide on how to use the open-source SLiCE can be found on GitHub [42].

#### Stratified Sampling by Clinical Concepts

Once the number of documents needed to create the gold standard as well as the numbers of positive and negative examples needed for the primary variables are calculated using SLiCE, the EHRs to be included in the final validation data set can be selected. In order to stratify clinical concepts, we need to collect the samples of each variable from the subset of documents from the target population. First, the positive and negative examples of the primary variable are selected according to SLiCE. In a second step, negative examples for secondary variables are randomly selected from reports excluding the primary variable. Positive examples for secondary variables are collected using a stratified sampling as a method of probabilistic sampling where the subgroups are identified by each secondary variable to ensure the representativeness of each linguistic event.

### Phase 3: Design of the Annotation Task and Guidelines

The preparation of the annotation project requires the cooperation of NLP experts and the internal medical experts (developers of the study). The annotation task itself is a manual process in which annotators (external medical experts of the participating hospital) review and mark up the predefined variables in the text for each EHR of the gold standard. To guarantee the quality of the resulting annotations [46], it is important to carefully design both the annotation guidelines and the annotation task.

The annotation guidelines consist of a set of instructions that explain what exactly the annotation task consists of. For instance, these guidelines will include the list of variables the annotators are expected to annotate in the free text, as well as resolve possible doubts related to, for example, synonyms or the inclusion of negative concepts. The creation of the guidelines is an iterative process in which NLP experts and internal medical experts participate. Using the initial draft of the annotation guidelines, the annotators are required to perform the annotation task on a small subset of documents in order to validate the design of the annotation project and correct, when applicable, the guidelines. This iterative process ensures that the instructions are clear before the start of the actual annotation task. The final guidelines need to be followed by each participating annotator in order to assure the consistency of annotations, especially across participating institutions. The process described here must always be applied, regardless of the study, the annotation tool (we use Inception at Savana [47]), or the number of documents included in the evaluation.

### Phase 4: External Annotations and Gold Standard Creation

Once the annotation project is prepared for each hospital participating in the study, the external annotation task can start. In this phase, 2 annotators (external medical experts) from each hospital will review independently and blindly (meaning they do not know which document they are annotating compared to their colleague) the whole set of documents selected in the previous steps. It is important to note that the 2 annotators are not allowed to communicate with each other or with the annotation project creators. Their only source of information are the annotation guidelines.

Once all the annotations have been completed by both annotators, a curator (additional external clinical expert) from that same institution is assigned to check every annotation for which the annotators disagree and to make the final decision. This final decision will be the one used for the gold standard creation that later serves to evaluate the cNLP system, while the two previous annotations are used to measure the interannotator agreement (IAA). The IAA is a commonly used approach in cNLP system evaluations [48-50] to identify the upper performance level.

### Phase 5: NLP Performance Evaluation

To measure the quality of annotations and to obtain target metrics for the cNLP system, it is necessary to assess them by measuring the IAA after full completion of the annotation task by the external medical experts. In our methodology, the IAA is calculated using the F1-score [51]. A low agreement can indicate that the annotators might have had difficulties in linguistically identifying the respective variables in the EHRs or that the guidelines are still inadequate in properly describing the annotation task. Thus, the IAA serves as a control mechanism to check the reliability of the annotation and further to establish a target of performance for the cNLP system.

The performance evaluation of the cNLP system is calculated using the standard metrics precision, recall, and their harmonic mean F1-score [43]. P gives us an indicator of the accuracy of information retrieved by the system (equation 3), R gives us an indicator of the amount of information the system retrieves (equation 4), and the F1-score gives us an overall performance indicator of information retrieval (equation 5):

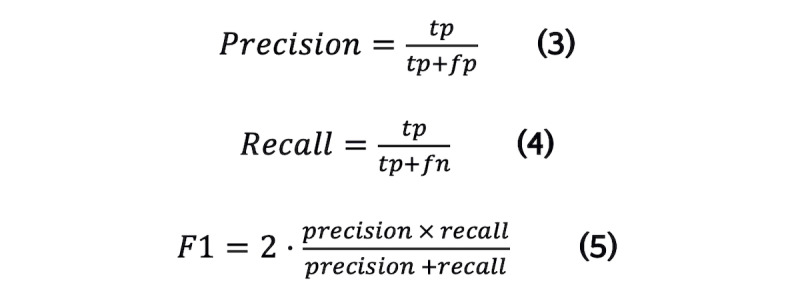



In all cases, true positives are the sum of records correctly retrieved, false negatives are the sum of records not retrieved, and false positives are the sum of records incorrectly retrieved.

In addition to these metrics, the 95% CI for each aforementioned measure can be calculated since this provides information about the range in which the true value lies and thus how robust the metric is. The method employed to calculate the CI is the Clopper-Pearson approach [45], one of the most common methods for calculating binomial CI.

## Results

### Application of the Methodology

The proposed evaluation methodology has been applied for the evaluation of cNLP systems in several clinical research projects at Savana. In this section, we give one example of its application in a project aimed at estimating the prevalence of severe asthma in the Spanish hospital population using Savana’s cNLP system “EHRead Technology”.

### Phase 1: Definition of the Target Population

For this study, the population was defined by adult patients with asthma (the primary medical condition), with EHRs available from multiple hospitals and a study period of several years.

### Phase 2: Statistical Document Selection Using SLiCE

#### SLiCE

With an average internal frequency for asthma of 48.5% in the target population of the participating hospitals (see subsection “Phase 2: Statistical Document Collection Using the Sample Size Calculator for Evaluations” of the Methods section for details on how this was calculated), an expected precision of 85% and recall of 80% (to be on the safe side, as explained in the previous section) with an interval width of 5% and expected CI of 95%, we obtained the following sample sizes:

- 249 positive examples;
- 270 negative examples;
- 519 total number of documents to annotate;
- 87 documents per hospital (42 positive examples, 45 negative examples).

#### Stratified Sampling by Clinical Concepts

In order to ensure the representativeness of the secondary variables of interest to the study, a stratifying approach was applied as explained in the subsection “Phase 2: Statistical Document Collection Using the Sample Size Calculator for Evaluations” of the Methods section (Table 1).

**Table 1:** Study variables detected in selected documents by the clinical natural language processing (cNLP) system compared to the ones obtained by manual annotations.

Variable	Manual annotations	cNLP system detections
Asthma (primary variable)	281	289
Extrinsic asthma	65	49
Bronchodilation test	131	88
Eosinophils in blood	181	164
Gastroesophageal reflux syndrome	181	168
Obesity	54	50
Omalizumab	27	21
Prick test	162	152
Salmeterol + fluticasone	147	80
Total IgE^a^	106	104

^a^IgE: immunoglobulin E.

### Phase 3: Design of the Annotation Task and Guidelines

External medical experts (annotators) were asked to mark the appearance of the clinical variables of interest in the free text of EHRs selected for the gold standard. In this project, we used the annotation tool Inception to facilitate the annotation task [47]. The annotation guidelines can be stored in this annotation tool, and annotators can access them via the user interface at any time during the annotation task.

### Phase 4: External Annotations and Gold Standard Creation

A crucial indicator is the IAA, which describes the difficulty of the external medical experts in evaluating the variables in the free text of EHRs and to set the target for the cNLP system performance. In the asthma study, the validation task appeared to be difficult as suggested by the suboptimal IAA F1-scores of several variables (Table 2). It was noted that the primary variable (asthma) and the first secondary variable (extrinsic asthma) may intersect, leading to confusion among medical experts. Once both annotators finished their annotations and the IAA was calculated, a third external medical expert resolved disagreements for the creation of the gold standard.

**Table 2:** Interannotator agreement (IAA) F1-scores for the primary and secondary variables of the annotation task.

Variable	IAA F1-score (95% CI)
Asthma (primary variable)	0.77 (0.70-0.82)
Extrinsic asthma	0.76 (0.58-0.88)
Bronchodilation test	0.86 (0.78-0.92)
Eosinophils in blood	0.68 (0.57-0.76)
Gastroesophageal reflux syndrome	0.82 (0.73-0.89)
Obesity	0.74 (0.51-0.87)
Omalizumab	0.88 (0.74-0.95)
Prick test	0.72 (0.62-0.80)
Salmeterol + fluticasone	0.81 (0.71-0.88)
Total IgE^a^	0.60 (0.45-0.72)

^a^IgE: immunoglobulin E.

### Phase 5: NLP Performance Evaluation

After the curation of the disagreements between the annotations of the external medical experts, the final gold standard was compared to the cNLP system, leading to higher precision and recall than expected for the primary variable and a CI width of 90-96 for precision and 94-98 for recall. The expected precision and recall used in SLiCE were underestimated compared to the final metrics, which means that even fewer reports could have been annotated. However, using as little as 519 EHRs, it was possible to evaluate the performance of the cNLP system and obtain performance for the primary variable within the expected CI range (Table 3). Interestingly, the performance metrics of the secondary variables were also high (>0.79) apart from one variable (total immunoglobulin E: F1=0.64) for which the IAA was also low (0.60).

**Table 3:** Performance metrics for primary and secondary variables when comparing the clinical natural language processing system to the gold standard.

Variable	Precision (95% CI)	Recall (95% CI)	F1 value (95% CI)
Asthma (primary variable)	0.94 (0.90-0.96)	0.96 (0.94-0.98)	0.95 (0.92-0.97)
Extrinsic asthma	1.00 (0.93-1.00)	0.75 (0.63-0.85)	0.86 (0.75-1.00 )
Bronchodilation test	0.99 (0.94-1.00)	0.66 (0.58-0.74)	0.79 (0.71-0.85)
Eosinophils in blood	0.99 (0.96-1.00)	0.90 (0.84-0.94)	0.94 ( 0.90-0.97)
Gastroesophageal reflux syndrome	1.00 (0.98-1.00)	0.93 (0.88-0.96)	0.96 (0.93-1.00)
Obesity	1.00 (0.93-1.00)	0.93 (0.82-0.98)	0.96 (0.87-1.00)
Omalizumab	1.00 (0.84-1.00)	0.78 (0.58-0.91)	0.93 (0.68-1.00)
Prick test	0.95 (0.91-0.98)	0.90 (0.84-0.94)	0.92 (0.87-0.96)
Salmeterol + fluticasone	0.98 (0.91-1.00)	0.53 (0.45-0.61)	0.96 (0.60-0.76)
Total IgE^a^	0.64 (0.54-0.74)	0.63 (0.53-0.72)	0.64 (0.54-0.73)

^a^IgE: immunoglobulin E.

## Discussion

### Principal Findings

We developed an easy-to-follow evaluation methodology, based on our experience with cNLP system evaluations using real-world clinical data, to provide guidance on how to evaluate their performance [36,38]. Our motivation was to be able to assure the robustness and representativeness of the performance metrics of evaluations of our cNLP systems, which is crucial for their application in clinical research. We presented the application of our evaluation methodology on a named-entity recognition cNLP system; however, the methodology can easily be adapted to other NLP tasks by adjusting the questions in phase 1 to the area of the respective NLP system. We routinely apply this methodology in our real-world evidence clinical studies and hope that it is equally useful for other NLP experts to reach a statistically sound evaluation of their own cNLP/NLP systems.

The first phase, the definition of the target population, is crucial for a successful evaluation [35] since it establishes the requisites for sampling and, most importantly, the scope of the cNLP system evaluation. While the linguistic characteristics to be considered for an evaluation are obvious, the questions needed to define the nonlinguistic characteristics are less obvious and require the insights of medical experts. Not properly defining the target population may lead to false expectations [37], a situation that can be avoided by initializing the cNLP system evaluation answering those questions. In our example, the definition of the target population was straightforward, but depending on the primary medical condition to be studied, this can be much more complex [52].

To ensure that the information extracted by a cNLP system is reliable and accurate, its output must be validated against a corpus of expert-reviewed clinical notes in terms of precision and recall of extracted medical terms. Thus, phase 2 of the evaluation methodology applied SLiCE, a statistical tool that offers guidance for the determination of a gold standard’s minimum sample size to ensure the expected levels of CIs of the linguistic events in cNLP system evaluations. The resulting gold standard contains a representative set of EHRs [35] based on the SLiCE output for the main variable in combination with the stratifying approach for the secondary variables, which has shown to lead to a much more representative gold standard than simple random sampling [35]. Frequently, evaluations are carried out using reference standards that are too small to be statistically useful, which might be due to limited resources [53], or that apply, at the other end of the extreme, a resource-wasting “the more, the better” approach [54]. Both scenarios are not satisfactory, and the use of SLiCE can help to avoid them without compromising the robustness of the evaluation or wasting resources.

In situations where the data source lacks predefined categories, other techniques such as discriminant text selection could be applied to limit the population from which to sample (eg, classifying EHRs into clinical services or departments). Thus, the frequency of the primary variable could be calculated over the category of interest, thereby increasing its frequency and lowering the amount of documents to be annotated. In phase 3, the annotation project is prepared with the input of both internal medical experts as well as NLP experts. NLP experts are heavily involved in many aspects of cNLP system evaluations as project leaders, consultants, technical support, providers of performance metrics, and most importantly, creators of the NLP system itself. But generally, NLP experts are not involved in the actual annotation task due to their lack of medical expertise. Nevertheless, the involvement of NLP experts early on in the creation of the annotation project to assist internal medical experts (eg, in the preparation of the guidelines or any NLP preprocessing) can be crucial for the final outcome of the annotation task [52,53].

To assure the quality of annotations and to provide a target for the expected accuracy of the cNLP system, we proposed the calculation of the IAA using the F1-score [51] in phase 4. Although other studies apply the Cohen kappa to measure IAA of mandatory and conditional questions [55], we preferred not to use kappa due to the lack of generalization [56]. Despite the debate about whether IAA really sets the upper limits for an cNLP system [56], we consider IAA to be important information to judge the performance of a cNLP system. In our use-case example, the cNLP system did not perform well for variable “Total IgE”; additionally, the annotators seemed to have had issues as revealed by the low IAA. This confirms the usefulness of the IAA to evaluate the difficulty of the identification of some variables and hence to better interpret the performance of the cNLP system.

Finally, in phase 5, the cNLP system performance is evaluated. In our use-case study of asthma patients, the performance was actually higher than expected. Although the gold standard could have been even smaller, the amount of documents to be annotated was close to the minimum to still assure the representativeness of the gold standard and robustness of the cNLP system evaluation. As mentioned in the Methods section, Phase 2 subsection, we suggest being conservative with SLiCE’s parameters to assure robust performance metrics. When presenting the evaluation results, mentioning the CI width for all performance measures is one of the criteria already defined by Friedman and Hripcsak [37] in their guidelines to improve cNLP system evaluations in the clinical domain. We follow this recommendation and advise every NLP expert to provide the upper and lower CI limits between which the true value lies, so that the robustness of the results can be determined.

### Limitations

Although our methodology offers a thoughtful strategy for cNLP system evaluations that has proven to be very useful, we want to point out some of its limitations. In several steps the methodology requires information that might not be easy to obtain in any project. If an NLP expert does not have access to internal medical experts to jointly work on the project, some of the required information might be difficult to obtain. In this case, external medical experts participating in the study would need to provide this information. In addition, the creation of the annotation project, including the annotation task and guidelines, requires both medical expertise and experience with annotation tasks to anticipate problems that nonexperienced annotators might encounter during the annotation task [53]. However, not all cNLP systems detect variables that can only be annotated correctly by medical experts. Depending on the level of medical knowledge required, and if no medical experts are available, nonexpert annotators can be recruited. Although considered a nonoptimal solution, nonexperts have successfully annotated text corpora in other projects [57-59].

Another problem might be the time required for the annotation task, which needs to be considered in the planning. With a primary variable of low frequency, the amount of documents to annotate can be quite high and external medical experts might not have the time to finish the annotation task in a timely manner or may even become upset with the annotation effort. Therefore, it is important to integrate medical expert knowledge to make sure that all nonlinguistic characteristics are covered to adjust the gold standard in the best possible way. To summarize, many aspects of a successful cNLP system evaluation in the clinical domain result from the essential collaboration between NLP experts and medical experts. The presented evaluation methodology reflects this important collaboration.

### Conclusion

We presented an evaluation methodology to guide NLP experts in cNLP system evaluations. By applying this methodology in a real study, we showed that this methodology is robust and efficient. To base the creation of the gold standard on performance metrics, results in a statistically useful gold standard which is a huge improvement over studies that do not base their decision on statistical measures. NLP experts who implement such internal controls in their cNLP system evaluation provide a robust evaluation and further respect medical experts’ time and economic resources.

## Appendix

Multimedia Appendix 1 The SLiCE algorithm.

## Abbreviations

cNLP: clinical natural language processing
EHR: electronic health record
IAA: interannotator agreement
NLP: natural language processing
P: precision
R: recall
SLiCE: Sample Size Calculator for Evaluations
