# Supplementary material for: Assessing the Performance of Clinical Natural Language Processing Systems: Development of an Evaluation Methodology
Source: JMIR Med Inform. 2021 Jul 23;9(7):e20492. doi: 10.2196/20492 (PMC8367121; doi:10.2196/20492)
Supplement: Multimedia Appendix 1 [file medinform_v9i7e20492_app1.docx]

**SLiCE: *S*amp*L*e s*i*ze *C*alculator for *E*valuations**

**Standard Performance Metrics**

Precision (*P*), Recall (*R*), and F1-score are standard metrics in evaluations of the Information Extraction (IE) system [[1]](https://www.zotero.org/google-docs/?HrTB88). *P* is an indicator of the accuracy of the information the system retrieves. R is the indicator of the amount of information the system retrieves.

Formally,

$P=\frac{tp}{tp+fp}$

$R=\frac{tp}{tp+fn}$

where

tp (true positives) = sum of cases correctly identified by the system,

fp (false positives)= sum of cases incorrectly identified by the system, and

fn (false negatives) = sum of cases not retrieved by the system.

F1-score gives us an overall performance indicator of information retrieval that combines *P* and *R* into a single metric through a harmonic mean of *P* and *R*:

$F1-score=2\cdot\frac{P\cdot R}{P+R}$

The harmonic mean is used instead of a simple average because it penalizes extreme values.

**Sample Size for a proportion**

*P* and *R* metrics are proportions. Thus, a method of sample size determination [[2]](https://www.zotero.org/google-docs/?RjRA0G) is employed to define the sample size of the variables *P* and *R*. The method consists of fixing a confidence level and a confidence interval (CI) width to calculate the sample size required to achieve the desired CI. Low CI widths with a high confidence level involve larger sample sizes. However, a confidence level of 95% and an interval width of 10% should be suitable for most tasks. In this proposal, the Clopper-Pearson approach is employed for CI calculation [[3]](https://www.zotero.org/google-docs/?4SGOfZ) since it is a common method for calculating binomial CI. However, other approximations could be considered. Under the Clopper-Pearson approach, the lower and upper confidence limits are determined by:

$[\frac{r}{r+(n-r+1) * F_{1-\alpha/2;2(n-r+1),2r)}}, \frac{(r+1) * F_{1-\alpha/2;2(r+1),2(n-r):}}{(n-r)+(r+1) * F_{1-\alpha/2;2(r+1),2(n-r)}}]$ (1)

where *n* is the number of trials (sample size), *F* is the F-Snedecor distribution, *r* is the number of successes, and α is the significance level (eg, 5%).

Considering a confidence level of 95%, CI width of 10%, and expected values of *P* and *R*, it is possible to obtain the sample sizes required to satisfy the confidence level. This proposal can be easily implemented in the R statistical software with the libraries “binom” [[4]](https://www.zotero.org/google-docs/?2ZIpAp) and “binomSamsize” [[5]](https://www.zotero.org/google-docs/?hxrRrs) or with the Python library “statsmodels” [6]. The latter was used in our SLiCE open-source code available on github (<https://github.com/Savanamed/slice>).

**Sample Size for Precision and Recall**

The Clopper-Pearson method returns the total number of observations required to satisfy the CI. For instance, with an expected *P* of 90%, one would need 155 observations to build a 95% CI with a 10% width. The resulting CI would be 84.2% $\leq$ *P* $\leq$ 94.2%. However, one needs to know the number of tp and fp in these 155 observations to determine the sample size. The tp, fp, tn and fn are the values determined with the following formulas:

**True Positives**

${tp}_{p}=P\cdot\left( {tp}_{p}+{fp}_{p} \right)$

${tp}_{r}=R\cdot\left( {tp}_{r}+{fn}_{r} \right)$

where,

${tp}_{p}$ = tp of precision,

${tp}_{r}$= tp of recall,

P = expected precision,

R = expected recall

**False Positives**

${fp}_{p}=\left( 1-P \right)\cdot\left( {tp}_{p}+{fp}_{p} \right)$

${fp}_{r}=\frac{{tp}_{r}\cdot\left( 1-P \right)}{P}$

where,

${fp}_{p}$ = fp of precision,

${fp}_{r}$ = fp of recall,

**False Negatives**

${fn}_{p}=\frac{{tp}_{p}\cdot\left( 1-R \right)}{R}$

${fn}_{r}=\left( 1-R \right)\cdot\left( {tp}_{r}+{fn}_{r} \right)$

where,

${fn}_{p}$ = fn of precision,

${fn}_{r}$ = fn of recall,

**True Negatives**

At this point, an estimation of the frequency (F) of the linguistic event to evaluate is required. In this research, a linguistic event refers to a particular clinical concept mentioned in Electronic Health Records such as a disease, a symptom, or a sign. Knowing the total number of positive events and the expected frequency of events, the total sample size (tp+fp+tn+fn) can be estimated. Then, subtracting the tp, fp, and fn from the total sample size, one obtains the total number of tn.

The frequency could be determined internally (Internal) by calculating the occurrence rate of the linguistic event in our data with an existing NLP system or with linguistic search engines. Another option is to use a frequency value extracted from a research publication or another resource (External).

***Internal frequency***

In the case of using the internal option, the frequency is (tp+fp)/(tp+fp+tn+fn) and the tn of precision (${tn}_{p}$) and recall (${tn}_{r}$) are obtained as:

${tn}_{p}=max\left( \frac{\left( {tp}_{p}+{fp}_{p} \right)\cdot\left( 1-F \right)-F\cdot{fn}_{p}}{F},0 \right)$

${tn}_{r}=max\left( \frac{\left( {tp}_{r}+{fp}_{r} \right)\cdot\left( 1-F \right)-F\cdot{fn}_{r}}{F},0 \right)$

***External frequency***

For the external option, the frequency would be (tp+fn)/(tp+fp+tn+fn) and the tn of precision (${tn}_{p}$) and recall (${tn}_{r}$) are obtained as:

${tn}_{p}=max\left( \frac{\left( {tp}_{p}+{fn}_{p} \right)\cdot\left( 1-F \right)-F\cdot{fp}_{p}}{F},0 \right)$

${tn}_{r}=max\left( \frac{\left( {tp}_{r}+{fn}_{r} \right)\cdot\left( 1-F \right)-F\cdot{fp}_{r}}{F},0 \right)$

After these calculations, the sample sizes for precision and recall are obtained. SLiCE keeps the greater value to guarantee the CI in both metrics. Thus, at the end of the process, SLiCE indicates the number of positives (tp+fp) and negatives (tn+fn) observations required to ensure the CI for the linguistic event evaluated. By obtaining reliable metrics for *P* and *R*, no sample size is required for the F1-score since it is the combination of these two metrics.

**References**

[1 Baeza-Yates, Ricardo A. R-N Berthier. *Modern Information Retrieval*. 75 Arlington Street, Suite 300 Boston, MAUnited States: Addison-Wesley Longman Publishing Co., Inc. 1999.](https://www.zotero.org/google-docs/?VcsfUo)

[2 Lwanga SK, Lemeshow S, Organization WH. *Sample size determination in health studies: a practical manual*. World Health Organization 1991.](https://www.zotero.org/google-docs/?VcsfUo)

[3 Clopper CJ, Pearson ES. The Use of Confidence or Fiducial Limits Illustrated in the Case of the Binomial. *Biometrika* 1934;**26**:404–13. doi:10.2307/2331986](https://www.zotero.org/google-docs/?VcsfUo)

[4 binom package | R Documentation. https://www.rdocumentation.org/packages/binom/versions/1.1-1 (accessed 26 Mar 2020).](https://www.zotero.org/google-docs/?VcsfUo)

[5 binomSamSize package | R Documentation. https://www.rdocumentation.org/packages/binomSamSize/versions/0.1-3 (accessed 26 Mar 2020).](https://www.zotero.org/google-docs/?VcsfUo)

6 Seabold S, Perktold J. statsmodels: Econometric and statistical modeling with python. In: 9th Python in Science Conference. 2010.
